# Cannabinoid Type 2 (CB_2_) Receptors Activation Protects against Oxidative Stress and Neuroinflammation Associated Dopaminergic Neurodegeneration in Rotenone Model of Parkinson's Disease

**DOI:** 10.3389/fnins.2016.00321

**Published:** 2016-08-02

**Authors:** Hayate Javed, Sheikh Azimullah, M. Emdadul Haque, Shreesh K. Ojha

**Affiliations:** ^1^Departments of Biochemistry, College of Medicine and Health Sciences, United Arab Emirates UniversityAl Ain, UAE; ^2^Department of Pharmacology and Therapeutics, College of Medicine and Health Sciences, United Arab Emirates UniversityAl Ain, UAE

**Keywords:** AM630, β-caryophyllene, cannabinoid agonist, neurodegeneration, neuroprotection, Parkinson's disease, rotenone, Trans-caryophyllene

## Abstract

The cannabinoid type two receptors (CB_2_), an important component of the endocannabinoid system, have recently emerged as neuromodulators and therapeutic targets for neurodegenerative diseases including Parkinson's disease (PD). The downregulation of CB_2_ receptors has been reported in the brains of PD patients. Therefore, both the activation and the upregulation of the CB_2_ receptors are believed to protect against the neurodegenerative changes in PD. In the present study, we investigated the CB_2_ receptor-mediated neuroprotective effect of β-caryophyllene (BCP), a naturally occurring CB_2_ receptor agonist, in, a clinically relevant, rotenone (ROT)-induced animal model of PD. ROT (2.5 mg/kg BW) was injected intraperitoneally (i.p.) once daily for 4 weeks to induce PD in male Wistar rats. ROT injections induced a significant loss of dopaminergic (DA) neurons in the substantia nigra pars compacta (SNpc) and DA striatal fibers, following activation of glial cells (astrocytes and microglia). ROT also caused oxidative injury evidenced by the loss of antioxidant enzymes and increased nitrite levels, and induction of proinflammatory cytokines: IL-1β, IL-6 and TNF-α, as well as inflammatory mediators: NF-κB, COX-2, and iNOS. However, treatment with BCP attenuated induction of proinflammatory cytokines and inflammatory mediators in ROT-challenged rats. BCP supplementation also prevented depletion of glutathione concomitant to reduced lipid peroxidation and augmentation of antioxidant enzymes: SOD and catalase. The results were further supported by tyrosine hydroxylase immunohistochemistry, which illustrated the rescue of the DA neurons and fibers subsequent to reduced activation of glial cells. Interestingly, BCP supplementation demonstrated the potent therapeutic effects against ROT-induced neurodegeneration, which was evidenced by BCP-mediated CB_2_ receptor activation and the fact that, prior administration of the CB_2_ receptor antagonist AM630 diminished the beneficial effects of BCP. The present study suggests that BCP has the potential therapeutic efficacy to elicit significant neuroprotection by its anti-inflammatory and antioxidant activities mediated by activation of the CB_2_ receptors.

## Introduction

The endocannabinoid system, consisting of cannabinoid receptors type 1 and 2 (CB_1_ and CB_2_), their endogenous ligands, and the enzymes for synthesis, reuptake and metabolism of the endocannabinoids, has emerged as an important neuromodulator system for many brain functions such as learning, memory, mood, addiction, and reward processing (Hill et al., 2009; Zanettini et al., 2011). Among the cannabinoid receptors, the CB_2_ receptor subtype has recently gained attention as an important therapeutic target for the modulation of neuroinflammation and attenuation of activated microglia and astrocytes in the substantia nigra and striatum (Bento et al., 2008; Concannon et al., 2015). The pharmacological activation of the CB_2_ receptors has been shown to reduce microglial activation and improve functional deficits in neurodegenerative diseases including Alzheimer's disease (Ramírez et al., 2005), Huntington's disease (Palazuelos et al., 2009; Sagredo et al., 2009), multiple sclerosis (Palazuelos et al., 2008), and PD (Price et al., 2009). In addition to the pharmacological studies, genetic studies also demonstrate enhanced microglial activation, neural pathology and inflammation in CB_2_ receptor knock-out mice (Palazuelos et al., 2008; Price et al., 2009). These studies indicated that cannabinoid-related compounds activating CB_2_ receptors may preserve neuronal homeostasis and survival in neurodegenerative disorders, including PD (Fernandez-Ruiz et al., 2007).

**Figure 1:**
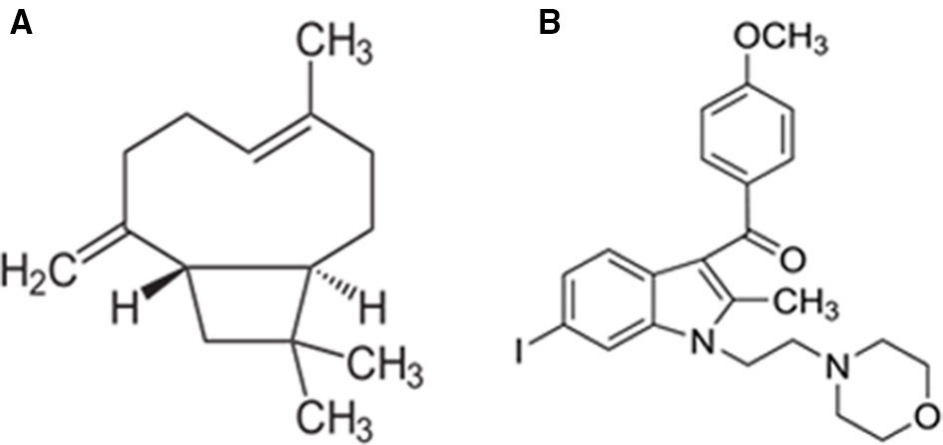
**Chemical structure of β-caryophyllene (A) and AM630 (B)**.

Furthermore, the CB_2_ receptor activation has been shown to be devoid of psychotropic, adverse effects, which are frequently observed with CB_1_ receptor modulation. Among the cannabinoid ligands, β-caryophyllene (BCP) generated enormous therapeutic interest due to its noteworthy identification as a fully selective agonist of CB_2_ receptors, its affinity and binding with CB_2_, receptors along with favorable physicochemical and pharmacokinetic properties (Figure 1; Gertsch et al., 2008). It is one of the widely available dietary phytocannabinoids and is commonly used as a preservative, additive, and flavoring in food and cosmetics. It has been recently added to the list of “generally regarded as safe” compounds for dietary use by the United States Food and Drug Administration (Gertsch et al., 2008). Chemically, BCP is a bicyclic sesquiterpene abundantly found in the essential oils of different species such as *Cinnamomum* spp. and *Piper* spp (Passos et al., 2004; Medeiros et al., 2007). BCP is a secondary metabolite predominantly found in many dietary plants that exhibits potent and long-lasting antioxidant and anti-inflammatory properties in different models of human diseases (Sharma et al., 2016). BCP is also an important constituent of *Cannabis sativa that* makes one of the major ingredients of Sativex, an approved drug for multiple sclerosis in European countries and Canada (Sibbald, 2005). BCP has been shown to elicit potent pharmacological properties such as anti-inflammatory (Gertsch et al., 2008), antioxidant (Singh et al., 2006), antispasmodic (Leonhardt et al., 2010), antidepressant and anxiolytic (Galdino et al., 2012; Al Mansouri et al., 2014), and anti-addictive (Bahi et al., 2014). Pharmacologically, it has been reported to be a CB_2_ receptor-selective agonist with a Ki value of 155 nmol/L for human CB_2_ receptors, with no affinity for CB_1_ receptors, which causes activation of Gi/Go subtype of G-proteins (Gertsch et al., 2008). The identification and participation of CB_2_ receptors in mediating neuroprotection has driven special interest in the pharmacological investigation of BCP for neurodegenerative disorders including PD.

Among the neurodegenerative diseases, PD is the second most common progressive disease. It is characterized by the selective degeneration of dopaminergic neurons in the substantia nigra pars compacta (SNpc) and depletion of dopamine neurotransmitter in the striatum (Shimohama et al., 2003). The pathogenesis of PD involves enhanced oxidative stress and mitochondrial dysfunction caused by the selective inhibition of the activity of complex I of the mitochondrial respiratory chain (Hu et al., 2010). The available evidence has shown that the decreased activity of the mitochondrial complex I has been observed in the SNpc of PD patients (Schapira, 1989) accompanied by increased oxidative/nitrosative stress (Jenner, 2003). The reactive oxygen species (ROS) generated from unregulated dopamine metabolism and auto-oxidation (Cadet and Brannock, 1998) further impair mitochondrial function (Tapias et al., 2009) and alter defensive endogenous antioxidant systems together leading to oxidative stress and contributing to progressive loss of dopaminergic neurons. Numerous studies have demonstrated that increased production of cytokines and ROS as well as induction of NADPH oxidase and inducible nitric oxide synthase (iNOS) leads to glial cells activation that contributes to PD pathology (Chen and Tansey, 2011). Therefore, interplay between the oxidative stress and subsequent neuroinflammation perpetuates neurodegeneration in PD (Lee et al., 2009).

Rotenone (ROT) induced rat model of PD was employed in the present study to investigate the therapeutic effects of BCP. ROT is a rotenoid pesticide used for understanding the pathogenesis of PD and evaluation of pharmacotherapeutic agents. It causes selective nigrostriatal degeneration, accumulation and aggregation of α-synuclein, oxidative stress, inflammation, microglial activation, abnormal locomotor activity, impaired motor functions, and disrupts mitochondrial complex I activity resulting in pathology strikingly similar to that observed in human PD patients (Betarbet et al., 2006; Cannon et al., 2009; Thakur and Nehru, 2013; Tapias et al., 2014). In the present study, we have investigated the neuroprotective effects of BCP in ROT induced rat model of PD, via CB_2_ receptor-dependent mechanism by using a selective CB_2_ receptor antagonist AM630 (Figure 1). AM630 (iodopravadoline) is a novel aminoalkylindole and a competitive cannabinoid receptor antagonist in the brain that is commonly employed as a pharmacological challenge to demonstrate the CB_2_ receptor dependent mechanisms (Hosohata et al., 1997). BCP has potential anti-inflammatory and anti-oxidant activity and is widely accessible for dietary intervention. We believe that our present findings may have important pharmacotherapeutic significance in the treatment/prevention of PD.

## Materials and methods

### Drugs and chemicals

Polyclonal rabbit anti-cyclo-oxygenase-2 (COX-2), anti-inducible nitric oxide synthase (iNOS), anti-glial fibrillary acidic protein (GFAP), anti-cannabinoid receptor type 2 (CB_2_) and anti-NF-kB p65 antibodies were purchased from Abcam, Cambridge, MA, USA. Anti-ionized calcium binding adaptor molecule-1 (Iba-1) polyclonal rabbit antibody was purchased from Wako Chemicals, Richmond, VA, USA. Polyclonal rabbit anti-tyrosine hydroxylase (TH) antibody was obtained from Novus Biologicals, Littleton, CO, USA. Alexa Fluor® 488 and 594 conjugated secondary goat anti-rabbit antibodies were purchased from Life Technologies, Grand Island, NY, USA. CB_2_ receptor antagonist, AM630 ([6-Iodo-2-methyl-1-[2-(4-morpholinyl) ethyl]-1*H*-indol-3-yl] (4-methoxyphenyl) methanone) was purchased from Tocris Bioscience, Ellisville, MO, USA. The lipid peroxidation kit for estimation of malondialdehyde (MDA) was obtained from North West Life science (Vancouver, WA, USA). The compounds: ROT, BCP and the assay kit for GSH were procured from Sigma-Aldrich, St. Louis, MO, USA. All the reagents used in the study were of analytical grade.

### Experimental animals

Six to seven months-old male Wistar rats (275–300 g), bred in the animal research facility of the College of Medicine and Health Sciences, United Arab Emirates University, were used. A maximum of four rats were housed per cage and were acclimatized for 1 week to the laboratory conditions prior to the start of the experiment. The animals were housed under standard laboratory conditions of 12/12 h of light and dark cycle. The animals had access to commercially available rodent food and water *ad libitum*. All the experiments were performed between 09:00 and 15:00 h. The experimental protocol for animal experimentation was approved by the Animal Ethics Committee of United Arab Emirates University, UAE.

### Experimental design

For the induction of PD in rats, ROT (2.5 mg/kg BW) was administered intraperitoneally (i.p.) once daily for 4 weeks. ROT was first dissolved in dimethyl sulfoxide (DMSO) at 50X stock solution and further diluted in sunflower oil to obtain a final concentration of 2.5 mg/ml. The regimen used in the current study for the induction of Parkinsonism in rats by ROT administration was described in a previous report (Ojha et al., 2015). To test the neuroprotective efficacy of CB_2_ receptor agonist, BCP, it was diluted in olive oil and administered i.p. at a dose of 50 mg/kg BW once daily for 4 weeks, 30 min prior to ROT administration in the presence or absence of AM630, a CB_2_ receptor antagonist. The dose of BCP was selected based on our previous study, in which a different set of experiments showed this dose to be pharmacologically optimal and devoid of any *in vivo* toxicity (Al Mansouri et al., 2014). The control group received a similar volume of vehicle. AM630 was dissolved in normal saline with 2.5% DMSO and tween 80 and injected 1 mg/kg BW i.p. 30 min prior to BCP treatment. The rats were divided into five groups as follows: Group I: Rats were injected vehicle only and abbreviated as control group (CONT), Group II: Rats were injected AM630 only and abbreviated as group AM630, Group III: Rats were injected rotenone dissolved in the vehicle and abbreviated as group ROT, Group IV: Rats were administered BCP, 30 min prior to rotenone injections and abbreviated as group ROT+BCP, Group V: Rats were administered AM630, 30 min prior to BCP treatment and rotenone and abbreviated as (ROT-BCP+AM630).

### Tissue preparation for biochemical studies

At the end of the 4 weeks, the animals were anesthetized with pentobarbital (40 mg/kg BW) and cardiac perfusion was performed using phosphate-buffered saline (PBS, 0.01 M, pH 7.4) to wash out the blood. The brains were quickly removed and placed on an ice plate to separate the two hemispheres. The midbrain and striatum were dissected from one hemisphere and immediately frozen in liquid nitrogen for further use. The other hemisphere was post-fixed first in paraformaldehyde solution (4%) for 48 h and subsequently in a series of sucrose solutions (10%) at 4⋅C changed three times a day for up to three consecutive days prior to cryostat sectioning.

### Biochemical studies

The midbrains of the rats collected from each group were homogenized in potassium chloride buffer at pH 8.0 (Tris-HCl 10 mM, NaCl 140 mM, KCl 300 mM, EDTA 1 mM, Triton X-100 0.5%) and supplemented with protease and phosphatase inhibitor. The tissue homogenates of the samples were centrifuged at 14000 g for 20 min at 4⋅C to obtain the post-mitochondrial supernatant (PMS) for estimation of antioxidant enzymes, lipid peroxidation product, and proinflammatory cytokines using spectrophotometric measurements and enzyme-linked immunosorbent assay (ELISA).

### Estimation of lipid peroxidation product

The extent of lipid peroxidation was measured by estimation of malondialdehyde (MDA). Briefly, the samples or calibrators (250 μl) were incubated in the presence of acid reagent and thiobarbituric acid (250 μl) and mixed vigorously using a vortex mixer. The samples were incubated for 60 min at 60⋅C and then centrifuged at 10,000 g for 2–3 min. The resultant reaction mixture was transferred to a cuvette and the absorbance was recorded at 532 nm. The results were expressed as μM MDA/mg protein.

### Estimation of GSH

The amount of GSH was estimated following the manufacturer's instructions for the commercially available kit. Briefly, the samples were first de-proteinized with 5-sulfosalicylic acid solution (5%) and centrifuged to remove the precipitated protein. The supernatant was used to estimate the amount of GSH. The samples or standards (10 μl) were incubated with 150 μl of working mixture (assay buffer with 5,5′-Dithiobis (2-nitrobenzoic acid) and glutathione reductase) in a 96-well plate for 5 min. Diluted NADPH solution (50 μl) was added to each well and mixed thoroughly. The absorbance of the samples was recorded at 412 nm using the microplate reader after 5 min incubation period. The results were expressed as μM GSH/mg protein.

### Antioxidant enzymes activity

The activities of antioxidant enzymes: superoxide dismutase (SOD) and catalase (CAT) were estimated using assay kits (Cayman Chemicals Co., Ann Arbor, MI, USA). Briefly, the activity of SOD was estimated by adding samples or standards (10 μl) to a 96-well plate. Xanthine oxidase (20 μl) was added to each well to initiate the reaction and the plate was shaken for a few seconds, afterwards it was covered and incubated for 30 min at room temperature. The absorbance was recorded using the microplate reader at 450 nm. The activity of CAT was estimated by adding samples or standards (20 μl) to the assay buffer (100 μl) and methanol (30 μl) in a 96-well plate. Hydrogen peroxide solution (20 μl) was added to initiate the reaction and incubated for 20 min at room temperature. Potassium hydroxide (30 μl) was added to terminate the reaction, subsequently catalase purpald (30 μl) and catalase potassium periodate (10 μl) were added. The plate was incubated for 5 min at room temperature on a shaker and absorbance was read at 540 nm using the microplate reader. The SOD activity was expressed as U/mg protein and the CAT activity was expressed as nmol·min^−1^·mg^−1^ protein.

### Estimation of nitrite levels

The nitrite levels were estimated using a commercially available kit (R&D system, Minneapolis, MN, USA). Briefly, the sample or nitrite standard (50 μl) was added with reaction diluent (50 μl) to a 96-well plate supplied by the manufacturer. Subsequently, Griess reagent (50 μl) was added to each well and mixed by gentle tapping on the side of the plate. The plate was incubated for 10 min at room temperature and the absorbance was measured at 540 nm using the microplate reader. The nitrite levels were expressed as μmol/mg protein.

### Estimation of proinflammatory cytokines

The levels of proinflammatory cytokines: interleukin-1β (IL-1β), interleukin-6 (IL-6), and tumor necrosis factor-alpha (TNF-α) were estimated using the commercially available ELISA kits purchased from R&D system (Minneapolis, MN, USA). Briefly, a 96-well plate was coated with the diluted capture antibody (100 μl) overnight at room temperature. Each well was aspirated and washed with the wash buffer (0.05% tween 20 in PBS 0.01M pH7.4). The plate was blocked by adding reagent diluent [containing 1% bovine serum albumin in PBS (300 μl)] for 1 h and washed with wash buffer. The samples or standards (100 μl) were added to the well and incubated for 2 h. The detection antibody (100 μl) was added to each well and then incubated for 2 h at room temperature. A working solution of streptavidin horseradish peroxidase (100 μl in ratio of 1:200) was added to each well and further incubated for 20 min. The contents of the wells were exchanged with substrate solution (100 μl) and incubated for 20 min. Stop solution containing 2N H_2_SO_4_ (50 μl) was added and the plate was gently tapped to ensure proper mixing. The absorbance of each well was measured immediately at 450 nm using a microplate reader. The results were expressed as pg/mg protein.

### Immunofluorescence staining of TH

The brain from each rat was collected as described above and sectioned for TH staining. Briefly, 14 μm coronal brain sections were cut at the levels of the striatum and the SNpc using a cryostat (Leica, Wetzlar, Germany). The sections were washed twice with PBS (0.01 M, pH 7.4) and then incubated with blocking reagent (10% normal goat serum in PBS, 0.3% Triton-X 100) for 1 h. Further, the sections were incubated with the primary polyclonal rabbit antibody against TH (1:500) overnight at 4⋅C. The sections were washed and incubated with fluorescent secondary antibody Alexa Fluor® 594 anti-rabbit (1:1000) for 1 h at room temperature. The sections were then washed and mounted using mounting medium Fluoroshield (Sigma Aldrich, MO, USA). Digital images of the sections were captured using a fluorescence microscope (Olympus, Hamburg, Germany).

### Immunofluorescence staining of GFAP and Iba-1

Immunofluorescence staining of the striatum was performed to examine the activation of GFAP positive astrocytes and Iba-1 positive microglia. Brain sections were washed twice with PBS and incubated with blocking reagent containing 10% normal goat serum and 0.3% Triton-X 100 in PBS for 1 h. The sections were then incubated with the primary polyclonal rabbit antibody against GFAP (1:1000) and Iba-1 (1:1000) overnight at 4⋅C. The sections were washed and incubated with fluorescent secondary antibody Alexa Fluor® 488 anti-rabbit for 1 h at room temperature. Sections were then washed and mounted using mounting medium Fluoroshield™. Images were captured using a fluorescence microscope, EVOS FL (Thermo Fisher Scientific, Waltham, MA, USA).

### Assessment of tyrosine hydroxylase-immunoreactive (TH-ir) dopaminergic (DA) neurons and TH-ir DA fibers

To determine the loss of the TH-ir neurons in the SNpc area, three different levels of the SNpc region (−4.8, −5.04, and −5.28 mm of bregma) were counted and the average was presented as a percentage. Loss of striatal fibers was evaluated by measuring the optical density of TH-ir DA fibers in the striatum (adjacent to 0.3 mm of bregma) using Image J software (NIH, Bethesda, MD, USA). For each rat, the optical density of TH-ir fibers within the striatum was measured in three different, equally sized fields of each section (three sections/rat). An average of the three areas was calculated and presented as a percentage with reference to the control. The optical density of the overlying cortex was taken as a background measurement and subtracted from the value generated from the striatum. The counting of TH-ir neurons and measurement of optical density of the TH-ir fibers were performed by an investigator blind to the experimental groups.

### Assessment of activated astrocytes and microglia

A minimum of three coronal sections, from each animal, at similar levels of the striatum were used to analyze the number of activated astrocytes and microglia. Activation of astrocytes and microglia was judged based on the intensity of the immunofluorescence and the presences of extended glial processes. Activated astrocytes and microglia were counted in three, randomly chosen, different, equally sized fields, using Image J software.

### Western blot analysis of NF-κB, COX-2, iNOS, and CB_2_

The striatal tissues from each experimental group were homogenized in the buffer to prepare the cytoplasmic and nuclear extracts as previously described by Arumugam et al. (2011). The samples of the cytoplasmic and nuclear fractions containing equal amounts of protein (35 μg) were separated by gel electrophoresis using SDS-polyacrylamide (10%). The proteins were transferred onto PVDF membrane and incubated overnight at 4⋅C with specific primary rabbit polyclonal antibodies against NF-κB p65 (1:500), COX-2 (1:1000), iNOS (1:500), and CB_2_ (1:500) followed by horseradish peroxidase-conjugated secondary anti-rabbit antibody. The protein recognized by the antibody was visualized using an enhanced chemiluminescence pico kit (Thermo Fisher Scientific, Rockford, IL, USA). The obtained blots were stripped and re-probed for β-actin (1:5000; monoclonal mouse, Millipore, MA, USA) as a loading control. The intensity of bands was measured by densitometry and quantified using Image J software.

### Estimation of protein concentration

The protein contents in the sample were estimated using the Pierce™ BCA protein assay kit (Thermo Fisher Scientific, Rockford, IL, USA) following the manufacturer's instructions.

### Statistical analyses

The data were expressed as the mean value ± SEM. The data for all parameters, unless otherwise stated, were analyzed using one-way analysis of variance (ANOVA), followed by Tukey's test to calculate the statistical significance between various groups, using software obtained from GraphPad InStat, La Jolla, CA, USA. In all the tests, the criterion for statistical significance was set at *p* < 0.05.

## Results

### BCP treatment upregulates expression of CB_2_ receptors

**Figure 2:**
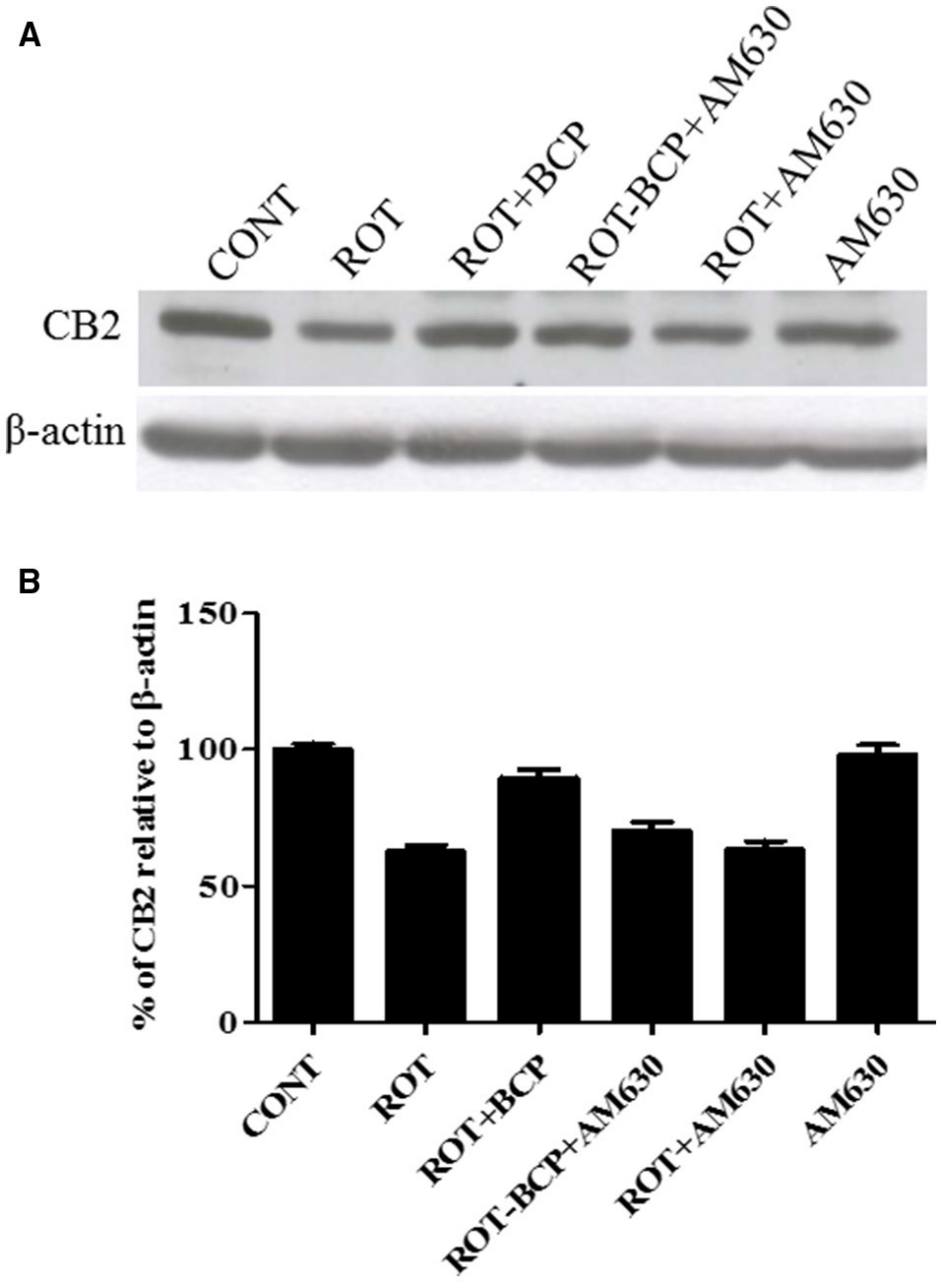
**The expression level of cannabinoid receptor 2 (CB_2_) measured by western blot in the striatum. (A)** The western blot showing CB_2_ expression. **(B)** CB_2_ expression was decreased in the ROT group as compared to the CONT group rats. However, BCP supplementation of ROT injected rats showed increased expression of CB_2_ receptors. Selective antagonist of CB_2_ receptors, AM630 administration prior to BCP supplementation decreased the expression of CB_2_ receptors. AM630 administration to ROT injected rats has not shown any noticeable difference in the expression of CB_2_. Finally, AM630 alone had no effect on the level of CB_2_ expression as compared to control rats. Values are expressed as percent (%) mean ± SEM (*n* = 3).

First, using western blot, we investigated, whether BCP treatment is capable of selectively enhancing the expression of CB_2_ receptors in the striatum region (Figures 2A,B). ROT administration decreased the expression of CB_2_ receptors as compared to CONT rats (62.56 vs. 100% control). BCP administration prior to ROT increased the expression of CB_2_ receptors in comparison to ROT-injected rats with no pretreatment (89.67 vs. 62.56%). However, AM630, a selective CB_2_ receptor antagonist, administered prior to BCP in ROT-injected rats caused a noticeable decline in the expression of CB_2_ receptors as compared to ROT+BCP group rats (70.22 vs. 89.67%). Further, we observed that AM630 administration to ROT-injected rats did not cause any change in the expression of CB_2_ receptors when compared to ROT-injected rats (63.28 vs. 62.56%). Additionally, the CONT and AM630-only groups of rats also did not show any marked differences in the expression level of CB_2_ receptors (98.19 vs. 100% control).

### CB_2_ receptor activation prevents loss of TH-ir DA neurons in SNpc and TH-ir DA fibers in striatum

**Figure 3:**
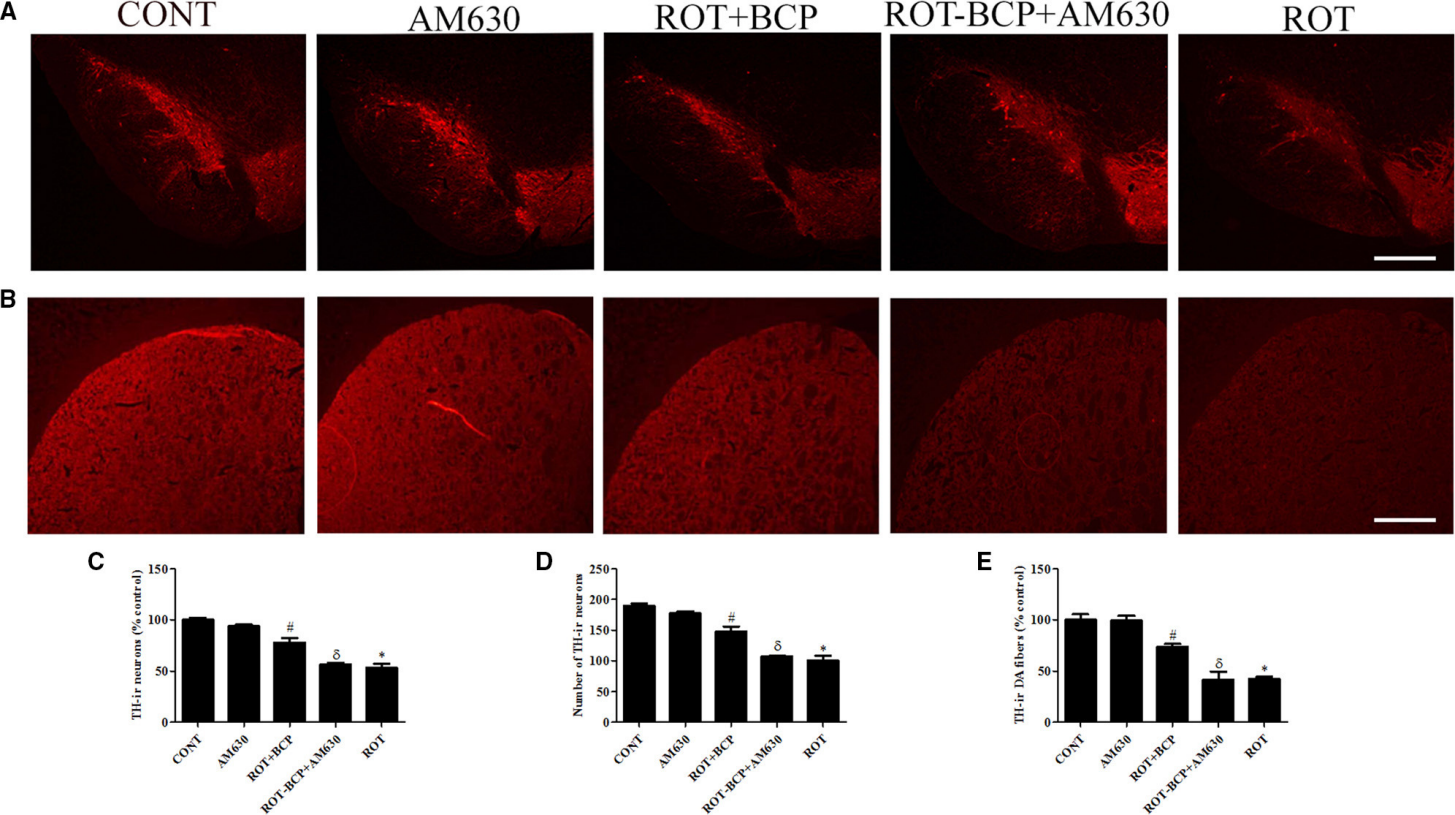
**The immunohistochemistry for tyrosine hydroxylase (TH) to detect the expression of the TH-immunoreactive (TH-ir) dopaminergic (DA) neurons in the substantia nigra pars compacta (SNpc) and the TH-ir dopamine nerve fibers in the striatum**. Values are expressed as percent mean ± SEM (*n* = 3). The scale bar is 100 μm. **(A)** The number of TH-ir neurons was decreased in the SNpc of ROT-injected rats as compared to control (CONT) group. While, BCP supplementation leads to increased expression of TH-ir neurons in ROT+BCP group as compared to ROT group. Furthermore, the ROT-BCP+AM630 group showed decreased expression of TH-ir neurons as compared to ROT+BCP group. **(B)** Similarly, TH-ir DA nerve fibers immunofluorescence was also decreased in the ROT group in comparison to the CONT group rats. Moreover, BCP supplementation increased the immunoreactivity of DA fibers as compared to ROT group. The ROT-BCP+AM630 group showed TH-ir DA fibers expression similar to ROT group. **(C,D)** The number of TH-ir DA neurons in the SNpc was counted from each group. A significant (*p < 0.05) decrease in the number of DA neurons was observed in the SNpc of the ROT group when compared to the CONT group. BCP treatment to ROT administered rats significantly (#*p* < 0.05) protected the DA neurons from the ROT-induced neuronal death in the ROT+BCP group. The ROT-BCP+AM630 group also showed significantly (^δ^*p* < 0.05) decreased number of DA neurons as compared to the ROT+BCP group. No significant differences were observed between the DA neurons of the CONT and AM630-only group. **(E)** Immunofluorescence of the TH-ir DA fibers was observed in the striatum of different groups. Significant (*p < 0.05) decrease in the TH-ir striatal DA fibers was observed in the ROT group as compared to the CONT group. Although, BCP supplementation of ROT treated rats significantly (#*p* < 0.05) inhibited the loss of the TH-ir DA fibers in the ROT+BCP group as compared to the ROT group. ROT-BCP+AM630 group showed significant (^δ^*p* < 0.05) loss of the TH-ir DA fibers when compared to the ROT+BCP group. CONT and AM630-only group did not show any apparent loss of TH-ir DA fibers.

To assess the neuroprotection afforded by BCP against the neurodegeneration caused by ROT, we determined the TH-ir DA neurons in the SNpc and optical density of TH-ir DA fibers in the striatum (Figures 3A–E). ROT administration to rats caused significant (*p* < 0.05) loss of DA neurons in the SNpc and striatal DA fibers in comparison with vehicle-injected control animals. However, BCP significantly (*p* < 0.05) prevented loss of DA neurons induced by ROT in the SNpc and striatal DA fibers compared to the ROT group of animals. Interestingly, this protective effect of BCP was significantly (*p* < 0.05) reversed by the prior administration of CB_2_ antagonist AM630 to rats administered with ROT and BCP. These results are indicative of the CB_2_ receptor-mediated neuroprotective effects of BCP. In contrast, AM630 administration alone did not show any deleterious effects on the DA neurons and striatal fibers.

### CB_2_ receptor activation attenuates the level of MDA and GSH in the midbrain

**Figure 4:**
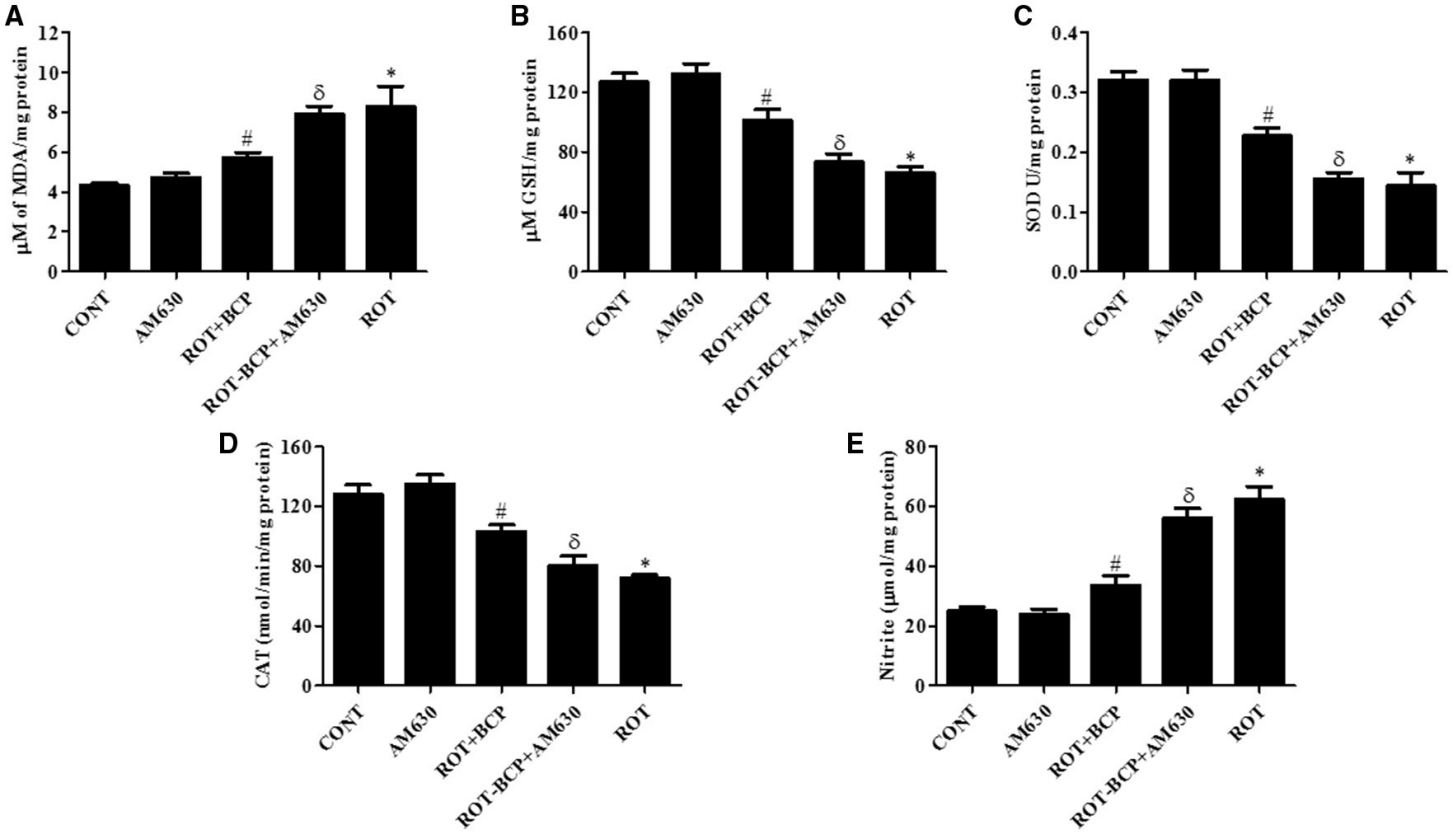
**The levels of malondialdehyde (MDA), glutathione (GSH), superoxide dismutase (SOD), catalase (CAT), and nitrite in the midbrain tissue**. Values are expressed as mean ± SEM (*n* = 6–8). ROT treatment caused significant (*p < 0.01) increase in MDA **(A)** and decrease in GSH level **(B)** in ROT challenged rats as compared to vehicle injected control (CONT) rats. BCP treatment of ROT challenged rats showed significantly (#*p* < 0.05) decreased level of MDA and increased (#*p* < 0.01) GSH level. However, CB_2_ antagonist AM630 administration prior to BCP treatment to ROT challenged rats significantly (^δ^*p* < 0.05) augmented the level of MDA and GSH as compared to ROT+BCP group rats. The activity of the antioxidant enzymes: SOD **(C)** and CAT **(D)** was significantly (*p < 0.05) decreased and nitrite **(E)** level was increased in the ROT group rats as compared to CONT group rats. BCP treatment of ROT administered ROT+BCP group rats significantly (#*p* < 0.05) augmented the SOD and CAT activity and nitrite level in comparison to the ROT group. ROT-BCP+AM630 group rats showed significant (^δ^*p* < 0.05) decline in SOD and CAT activity and rise in nitrite level as compared to ROT+BCP group rats.

ROT administration significantly (*p* < 0.01) increased MDA content (Figure 4A) and decreased (*p* < 0.01) the level of GSH (Figure 4B) as compared to control group animals. However, BCP pretreatment of ROT-administered rats caused a significant (*p* < 0.05) reduction in MDA level and prevented (*p* < 0.01) decline of GSH level. In contrast, in ROT-challenged rats, administration of AM630 before BCP significantly (*p* < 0.05) diminished the effect of BCP on MDA reduction and GSH restoration (Figures 4A,B). Interestingly, the AM630-only group did not show alteration in the level of either MDA or GSH as compared to control animals.

### CB_2_ receptors activation augments antioxidant enzymes activity in the midbrain

The activities of antioxidant enzymes: SOD (Figure 4C) and CAT (Figure 4D) was significantly (*p* < 0.05) decreased in ROT-injected rats compared to control rats. However, a significant (*p* < 0.05) increase in the activity of SOD and CAT was observed in BCP-treated rats as compared to ROT-injected rats. In contrast, AM630 administration diminished the effects of BCP in ROT-challenged rats (Figures 4C,D). The antioxidant enzyme activity of SOD and CAT was unaltered in the AM630-only group of animals.

### CB_2_ receptors activation inhibits increased nitrite level in the midbrain

The nitrite levels were significantly (*p* < 0.05) increased in the ROT-administered rats as compared to control rats (Figure 4E). However, treatment with BCP produced a significant (*p* < 0.05) decrease in the level of nitrite and that in turn was significantly (*p* < 0.05) attenuated by the CB_2_ receptor antagonist, AM630 (Figure 4E). In contrast, administration of CB_2_ receptor antagonist AM630-only did not cause any significant change in the nitrite level.

### CB_2_ receptor activation alleviates ROT-induced glial cell activation in the striatum

**Figure 5:**
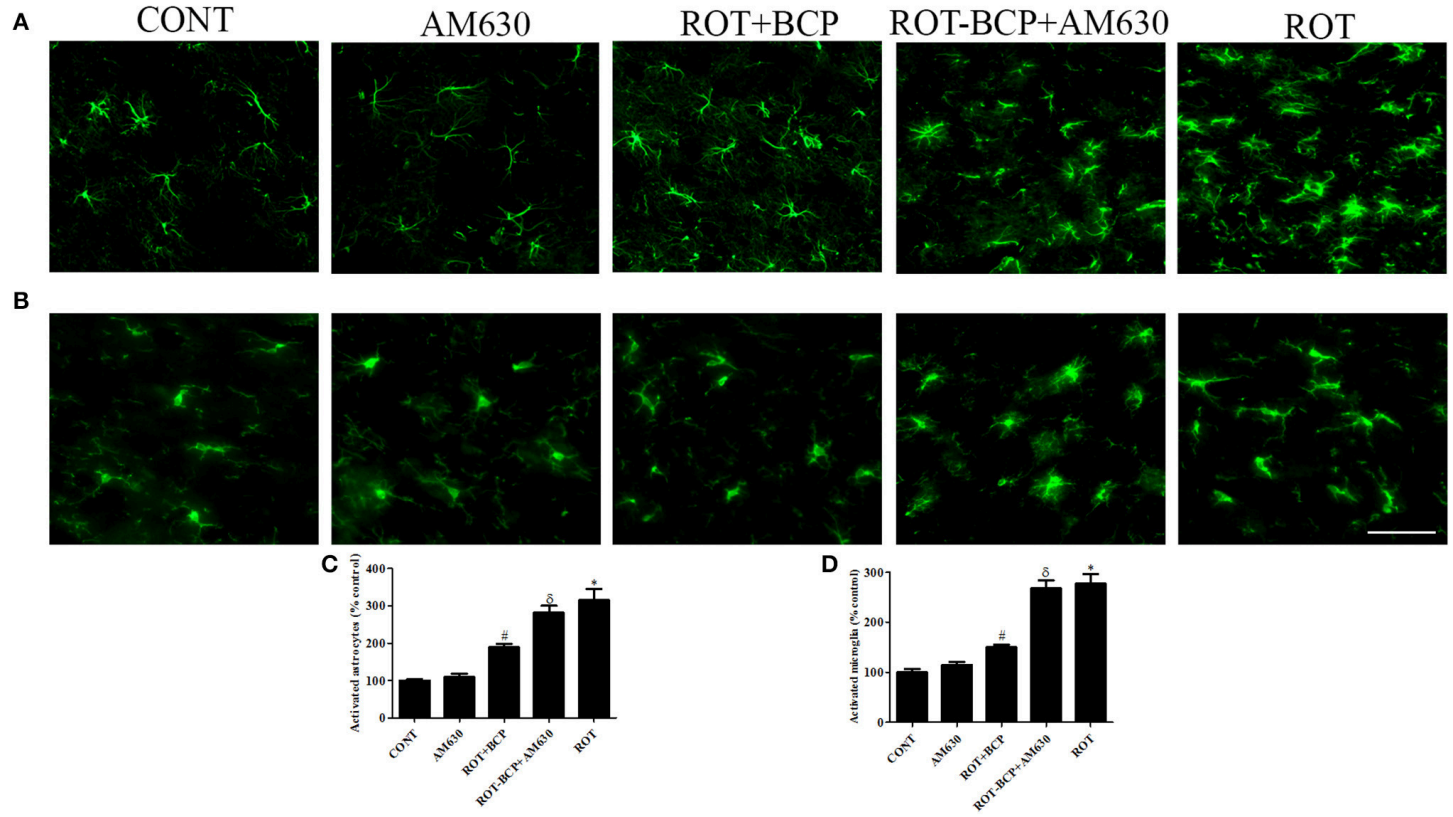
**The immunofluorescence staining to detect the expression of glial fibrillary acidic protein (GFAP) positive astrocyte and ionized calcium binding adaptor molecule-1 (Iba-1) positive microglia in the striatum**. Values are expressed as percent mean ± SEM (*n* = 3). The scale bar is 200 μm. Profound expression of GFAP positive astrocytes **(A)** and Iba-1 positive microglia **(B)** was found in the ROT-administered and ROT-BCP+AM630 groups as compared to the vehicle-injected CONT and AM630-only groups. While BCP supplementation to ROT-injected rats showed moderate staining of GFAP and Iba-1 as compared to ROT-injected rats. Quantitative analysis of activated astrocytes **(C)** and microglia **(D)** has shown that significant (*p < 0.05) increase in number of activated astrocytes and microglia was observed in the ROT group as compared to the CONT group. However, BCP supplementation significantly (#*p* < 0.05) reduced the number of activated astrocytes and microglia in the ROT+BCP group as compared to the ROT group. The ROT-BCP+AM630 group also showed significantly (^δ^*p* < 0.05) increased number of activated astrocytes and microglia when compared to the ROT+BCP group. The CONT and AM630-only groups did not show any marked difference in the activation of astrocytes and microglia.

Immunofluorescence staining of GFAP and Iba-1 was carried out to determine the morphological changes in astrocytes and microglia, respectively. In the ROT-administered rats, a noticeable rise in immunofluorescence of GFAP and Iba-1 was observed, which is indicative of the activation of astrocytes and microglia, the features observed in PD neurodegeneration. However, BCP treatment showed a comparatively decreased immunofluorescence of GFAP and Iba-1 (Figures 5A,B). Additionally, a quantitative evaluation for GFAP-positive astrocytes and Iba-1-positive microglia was performed in the rats of different experimental groups (Figures 5C,D). ROT-injected rats showed a significantly (*p* < 0.05) higher number of activated astrocytes and microglia compared to control animals, whereas BCP pretreatment of ROT-injected rats created a significant (*p* < 0.05) reduction in the number of activated astrocytes and microglia as compared to ROT-challenged rats. Further, AM630 administration prior to BCP supplementation to ROT injected animals showed increase in the number of activated astrocytes and microglia clearly demonstrating the CB_2_ receptor-mediated activity of BCP. However, AM630 alone did not have a significant effect on the activation or number of astrocytes and microglia.

### CB_2_ receptors improve the level of proinflammatory cytokines in the midbrain

**Figure 6:**
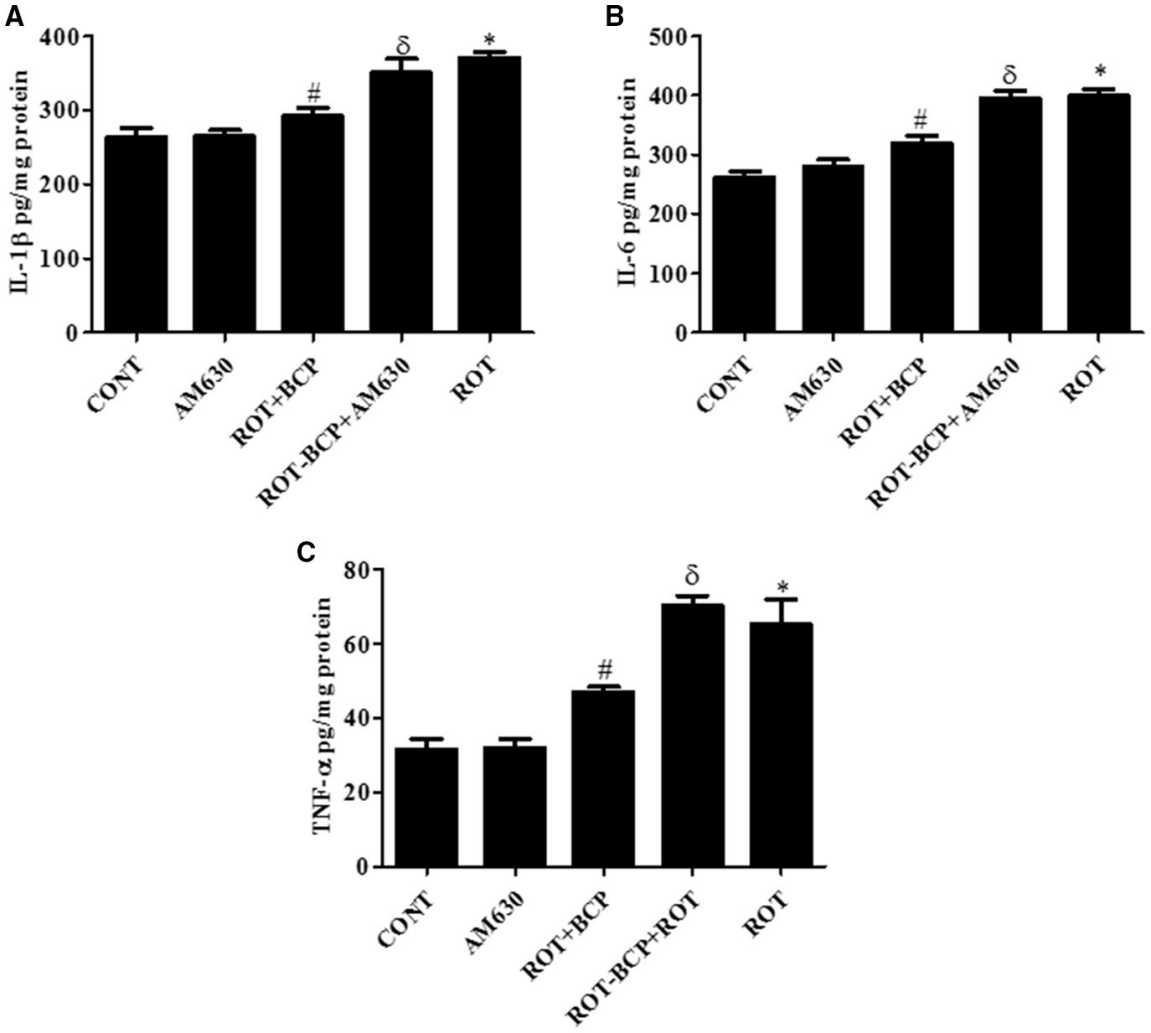
**The pro-inflammatory cytokines: IL-1β, IL-6, and TNF-α were measured by enzyme linked immunosorbent assay (ELISA) in the midbrain**. Values are expressed as mean ± SEM (*n* = 6–8). The level of IL-1β **(A)**, IL-6 **(B)**, and TNF-α **(C)** was significantly (*p < 0.05) increased in the ROT group when compared to the CONT group. Although, BCP supplementation significantly (#*p* < 0.05) decreased the ROT-induced increase of these proinflammatory cytokines in the ROT+BCP group. The ROT-BCP+AM630 group showed significantly (^δ^*p* < 0.05) increased level of IL-1β, IL-6, and TNF-α as compared to the ROT+BCP group. There is no significant difference in these cytokines between the CONT and the AM630-only group.

ROT administration created a significant (*p* < 0.05) increase in the level of proinflammatory cytokines such as IL-1β (Figure 6A), IL-6 (Figure 6B), and TNF-α (Figure 6C) as compared to the control group while treatment with BCP significantly (*p* < 0.05) attenuated the rise of these proinflammatory cytokines in ROT-injected rats (Figures 6A–C). However, AM630 administration before BCP abrogated the protective effects of BCP against ROT-induced elevated levels of proinflammatory cytokines. The CB_2_ receptor antagonist AM630 alone, did not have any effect on the level of proinflammatory cytokines.

### CB_2_ receptor activation attenuates inflammatory mediators NF-κB, COX-2 and iNOS

**Figure 7:**
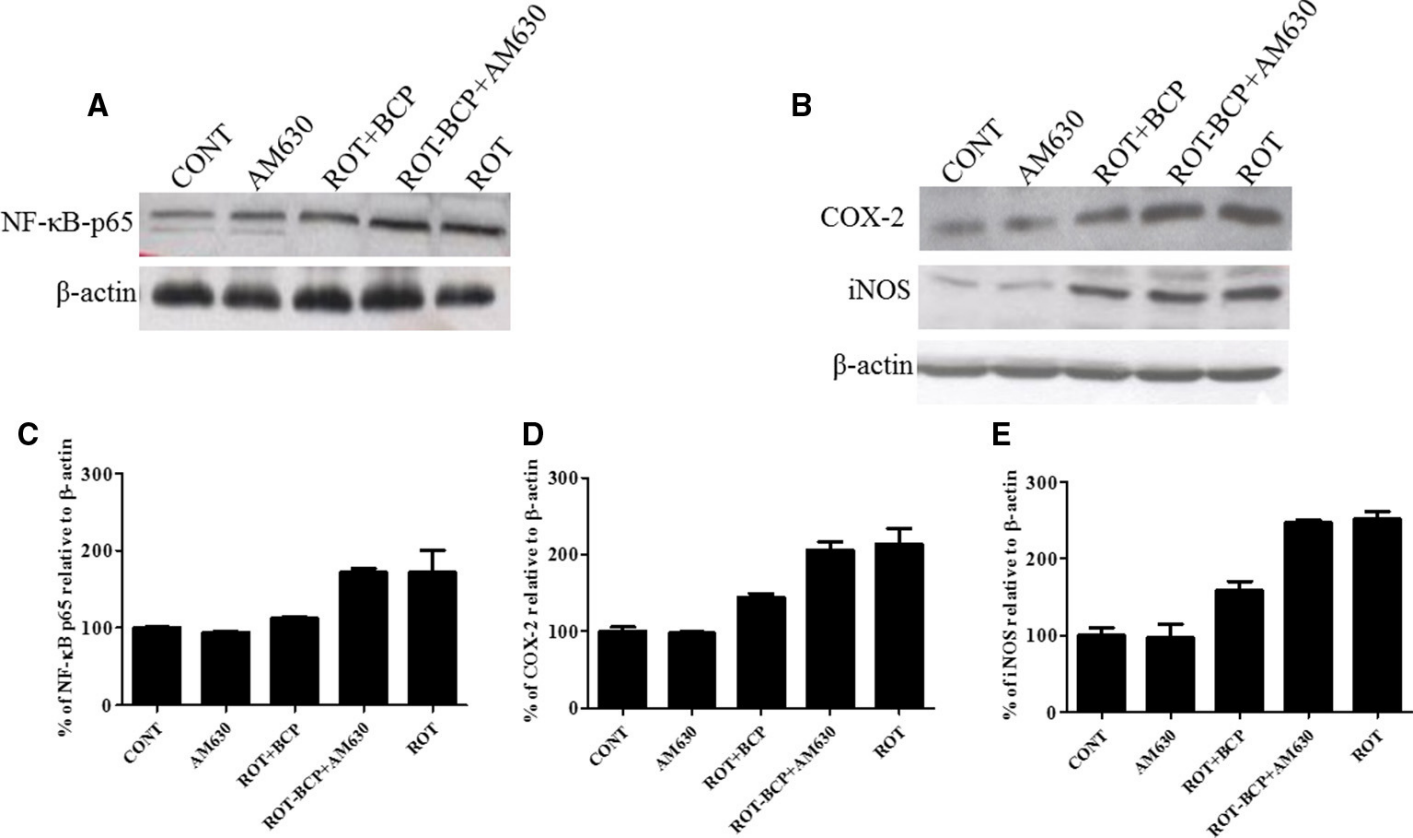
**Western blot analysis of NF-κB p65, COX-2, and iNOS in the striatal tissue**. Values are expressed as percent (%) mean ± SEM (*n* = 3). Expression level of NF-κB p65 **(A)** COX-2 and iNOS **(B)** was carried out by western blot. **(C)** ROT-administered group rats showed increase in nuclear NF-κB p65 level as compared to control group (171.7 vs. 100% control). BCP treatment of the ROT-challenged rats decreased the NF-κB p65 expression when compared to the ROT group (112.38 vs. 171.7%). The ROT-BCP+AM630 group had shown increased expression of NF-κB p65 as compared to the ROT+BCP group (171.55 vs. 112.38%). **(D)** ROT-injected rats showed increased COX-2 level as compared to the CONT rats (213.48 vs. 100% control). BCP supplementation to ROT-injected rats showed decreased expression of COX-2 as compared to ROT group rats (143.6 vs. 213.48%). ROT-BCP+AM630 group rats showed increase in COX-2 expression as compared to the ROT+BCP group rats (205.6 vs. 143.6%). **(E)** Likewise, iNOS expression was increased in the ROT group rats as compared to the CONT group rats (251.56 vs. 100% control). BCP treatment decreased iNOS expression as compared to the ROT group rats (158.12 vs. 251.56%). The ROT-BCP+AM630 group showed increase in iNOS expression when compared to the ROT+BCP group (247.18 vs. 158.12%).

To detect and quantify the activation of NF-κB, the translocation of NF-κB p65 subunit to the nucleus was assayed in the striatal nuclear extracts using a subunit specific anti-NF-κB p65 antibody. As depicted in Figures 7A,C, the rats injected with ROT showed a remarkably higher level of NF-κB p65 as compared to the controls (171.7 vs. 100% control). However, treatment with BCP to ROT-administered rats produced a decrease in NF-κB p65 levels as compared to the ROT group (112.38 vs. 171.7%). In contrast, the CB_2_ receptor antagonist AM630 administered prior to BCP in ROT-injected rats increased the level of NF-κB p65 expression as compared to the ROT+BCP group (171.55 vs. 112.38%).

We further investigated the protein expression level of NF-κB responsive genes such as COX-2 and iNOS in the cytoplasmic fraction of the striatal tissue (Figure 7B). Similar to NF-κB p65, the expression level of COX-2 and iNOS increased in ROT-challenged rats as compared to control rats (COX-2: 213.48 vs. 100% control; iNOS: 251.56 vs. 100% control). However, BCP pretreatment of ROT-challenged rats decreased the elevated level of COX-2 and iNOS as compared to ROT injected animals (COX-2: 143.6 vs. 213.48%; iNOS: 158.12 vs. 251.56%). However, AM630 administration before BCP has abrogated the beneficial effect of BCP against COX-2 and iNOS in animals treated with ROT (COX-2: 205.6 vs. 143.6%; iNOS: 247.18 vs. 158.12%) (Figures 7D,E). Similar to oxidative stress and morphological parameters, no significant changes in the levels of NF-κB, COX-2 and iNOS were observed in the AM630-alone group of rats.

## Discussion

The current therapies for PD are still inadequate. Therefore, intensive efforts are made to identify promising new targets for the treatment of PD. Until now, the exact cause of PD has yet to be established even though environmental factors, in addition to genetic factors, have been demonstrated to play a significant role in the causation of PD. Based on the environmental factors, the present study employed the ROT-induced rat model of PD that is widely used for the evaluation of novel pharmacotherapeutic agents and understanding of etiopathogensis. ROT has been shown to cause PD with the inhibition of mitochondrial complex I of the respiratory chain and activation of abnormal dopamine metabolism (Betarbet et al., 2000; Sherer et al., 2007), along with oxidative stress and inflammation, which causes neurodegeneration of DA neurons in the SNpc region of the PD brain (Johnson and Johnson, 2015). The results from the present study showed that ROT causes loss of dopaminergic neurons in the SNpc and the striatal dopaminergic nerve terminals. This correlates with the degeneration of the nigrostriatal pathway, which plays an important role in the co-ordination of motor functions. The degeneration of nigrostriatal pathways has been shown to be associated with the occurrence of oxidative/nitrosative stress, which subsequently leads to the induction of neuroinflammation and subsequent neurodegeneration (Hald and Lotharius, 2005; Cannon et al., 2009; Lee et al., 2009). In recent years, the cannabinoid receptors, specifically activating CB_2_ receptors, appear to represent a novel therapeutic target for neurodegenerative diseases, including PD, because of their role in counteracting oxidative stress and inflammation. The CB_2_ receptors have recently emerged as a potential anti-inflammatory target, to break the self-sustaining cycle of neuroinflammation and preserve neuronal homeostasis, and survival in neurodegenerative disorders (Ramírez et al., 2005; Palazuelos et al., 2008; Price et al., 2009; Sagredo et al., 2009; Gómez-Gálvez et al., 2016). The activity of CB_2_ receptors is implicated in the reduction of proinflammatory mediators in response to noxious stimuli and in control of neuronal survival (Fernandez-Ruiz et al., 2007). Recently, changes in CB_2_ receptors have been shown in the mice model of PD induced by intrastriatal lesions with lipopolysaccharides (García et al., 2011). Genetic studies have also shown that CB_2_ receptor knockout-mice display microglial activation, neural pathology, and functional deficits in the MPTP-induced mice model of PD (Price et al., 2009). The CB_2_ receptor expression has been demonstrated in the central nervous system, including the SNpc area of PD brains (García et al., 2015). A recent report also suggests that CB_2_ receptors modulate the dopamine dependent neuronal activity and behavior in mice (Zhang et al., 2014). Because of potent anti-inflammatory and antioxidant action, and no psychoactive adverse reactions caused by CB_2_ receptor activation, the therapeutic targeting of CB_2_ receptor appears to be promising for modulation of neuroinflammation and disease modification in PD.

In addition, ROT has been reported to inhibit complex I of the mitochondrial respiratory chain, which consequently leads to the formation of ROS, such as superoxide, hydroxyl radical and peroxynitrite. The induction of ROS is critical in the SNpc because of low levels of antioxidant defenses corresponding to a very high level of dopamine metabolism in this region (Thakur and Nehru, 2013). To determine antioxidant tissue defense, we assessed the oxidative/nitrosative stress parameters such as activities of antioxidant enzymes like SOD and CAT; and the levels of GSH, total nitrite, and MDA, in the midbrain tissues. The occurrence of lipid peroxidation is a key pathogenic event in tissues, resulting from an imbalance between ROS generation and the availability of endogenous cellular antioxidant defense. We observed that the ROT-challenge caused a significant rise in the MDA level, with a concomitant decline in the GSH level in the midbrain tissues. Interestingly, treatment with BCP of ROT-injected rats inhibited lipid peroxidation evidenced by the reduced MDA levels followed by restoration of the GSH content. The decline in the MDA content and concomitant restoration of GSH content by BCP can be ascribed to the potent chain breaking, free radical scavenging, and antioxidant activity of BCP (Chang et al., 2007).

Apart from non-enzymatic antioxidants, the cells are also equipped with the enzymatic defense system, which includes SOD and CAT, as an important component in neutralizing the ROS load. The present study showed that ROT caused a significant reduction in the activities of SOD and CAT enzymes as compared to control rats. However, BCP treatment of ROT-challenged rats showed a significant restoration of SOD and CAT activities. Furthermore, in ROT-challenged rats, we observed a significant rise in the total nitrite levels, due to the production of nitric oxide that generates peroxynitrite on reaction with superoxide anion leading to robust neurotoxicity (Beckman et al., 1990). BCP treatment of ROT-challenged rats significantly counteracted any rise in nitrite levels. The reversal of these beneficial effects of BCP against oxidative stress by prior administration of AM630, a selective CB_2_ receptor antagonist, clearly demonstrates CB_2_ receptor-dependent activity of BCP. To our knowledge, the present study is the first to report that BCP reduces oxidative stress in ROT-induced dopaminergic neurodegeneration by activating CB_2_ receptors.

Previous studies have demonstrated gliosis with increased expression of GFAP and Iba-1 in human PD brains that appears to be age dependent and related to the disease progression (Yan et al., 2014). The glial cells mediating neuroinflammation have recently emerged as key players in the pathogenesis of PD; and the activation of microglia in the nigrostriatal regions of the PD brains is believed to be a rapid cellular response to inflammation (Hirsch et al., 2003; Gordon et al., 2012). In our study, the hypertrophy with long processes, along with the increased immunofluorescence of GFAP and Iba-1, represents activated astrocytes and microglia, respectively. However, BCP treatment reduced the activation of astrocytes and microglia, which supports its anti-inflammatory action in-line with the reported activity of several other agonists of the CB_2_ receptors (Marchalant et al., 2008). Furthermore, it was expected that a CB_2_ receptor antagonist should reverse or abolish the protective effects of BCP, as indeed was the case, as the administration of AM630, abrogated the effects of BCP regarding glial cell activation. The findings clearly demonstrate a functional CB_2_ receptor dependent mechanism in the protective effects of BCP.

Moreover, microglia activation leads to the nuclear translocation of transcription factor NF-κB, which upregulates the release of inflammatory mediators such as COX-2 and iNOS as well as induction of proinflammatory cytokines such as IL-1β, IL-6, and TNF-α in PD (Mosley et al., 2006). The transcription factor NF-κB also plays an important role in the regulation of several proinflammatory mediators that are directly involved in the initiation of neuroinflammation and subsequent neurodegeneration. The inactive form of NF-κB is present in the cytoplasm in association with the inhibitory protein IκB, which prevents its nuclear translocation that is required for transcriptional activity. The IκB phosphorylation followed by proteolytic degradation results in the translocation of a free p65 subunit of NF-κB to the nucleus where it binds to target DNA elements (gene promoters containing κB binding sites) and regulates transcription of several proinflammatory genes such as iNOS, COX-2, IL-1β, IL-6, and TNF-α (Shen et al., 2010). These inflammatory enzymes (COX-2 and iNOS and proinflammatory cytokines IL-1β, IL-6 and TNF-α) are known to be cytotoxic to neurons and to lead to neuronal cell death (Hald and Lotharius, 2005). Numerous studies have reported that cannabinoid ligands inhibit the production of proinflammatory cytokines such as TNF-α, IL-1β, and IL-6; and suppress production of proinflammatory mediators by microglia and macrophages, by CB2 receptor modulation (Correa et al., 2005; Klein, 2005; Zhao et al., 2010).

Our study demonstrates that BCP inhibited the ROT-induced nuclear translocation of NF-κB p65, whereas administration of AM630 prior to BCP supplementation to ROT-injected rats diminished the beneficial effects of BCP. We also observed elevated expression of inflammatory mediators COX-2 and iNOS in ROT-challenged rats. BCP treatment of ROT-challenged rats modestly decreased the expression of COX-2 and iNOS, which supports its anti-inflammatory activity as reported by others (Cho et al., 2007; Bento et al., 2011). In contrast, the anti-inflammatory effect of BCP against increased expression of COX-2 and iNOS was abolished by prior administration of AM630. Additionally, proinflammatory cytokines (IL-1β, IL-6, and TNF-α) were also found significantly increased after ROT-challenge of the rats. BCP treatment significantly decreased the elevated levels of these proinflammatory cytokines in ROT-injected rats. In contrast, AM630 antagonized the protective effect of BCP against the proinflammatory cytokines. Taken together, these results demonstrate that the beneficial effects of BCP were reversed by AM630. To conclude, the abrogation of the BCP effects by AM630 clearly demonstrates that the activity of the central cannabinoid receptors plays an important role in mediating of the neuroprotective action of BCP in ROT-induced PD.

Further, the observed neuroprotective activity of BCP in the present study was also supported by a previous *in vitro* study, where BCP (among a group of triterpenoids screened for pharmacological activity) showed the highest neuroprotective effects due to favorable physicochemical properties, including high lipophilicity (Chang et al., 2007). The lipophilicity of BCP and AM630 are both more than four, which is strongly indicative of their ability to permeate the blood brain barrier and of their bioavailability in the brain. The CB_2_ receptor antagonist AM630 is a protean ligand and inverse agonist that targets a constitutively active form of CB_2_ receptor in the brain (Bolognini et al., 2012). The CB_2_ receptor ligands are generally classified as agonists, antagonists and inverse agonists showing positive, neutral and negative efficacy, respectively, for a particular signaling pathway. They show varying potency and efficacy termed as “functional selectivity” or “biased agonism” (Pertwee et al., 2010), and pave the way to the design of agents, which may activate the specific pathways required for therapeutic benefit. The abrogation of CB_2_ receptor mediated effects by AM630 demonstrates the CB_2_ receptor dependent mechanism and has been shown in a number of studies (Hosohata et al., 1997).

The data in our study is in agreement with previous studies of the action of BCP and AM630 at CNS level (Choi et al., 2013). The previous report of the effects of BCP in enhancing cognition, preventing dependence and alleviating anxiety, depression, and compulsion is further supported by our current findings (Al Mansouri et al., 2014). Taken together, the abrogation of the protective effects of BCP by AM630 demonstrates the CB_2_ receptor-dependent mechanism of BCP and the findings can be extrapolated to the neuroprotective properties of CB_2_ agonism in PD.

## Conclusion

**Figure 8:**
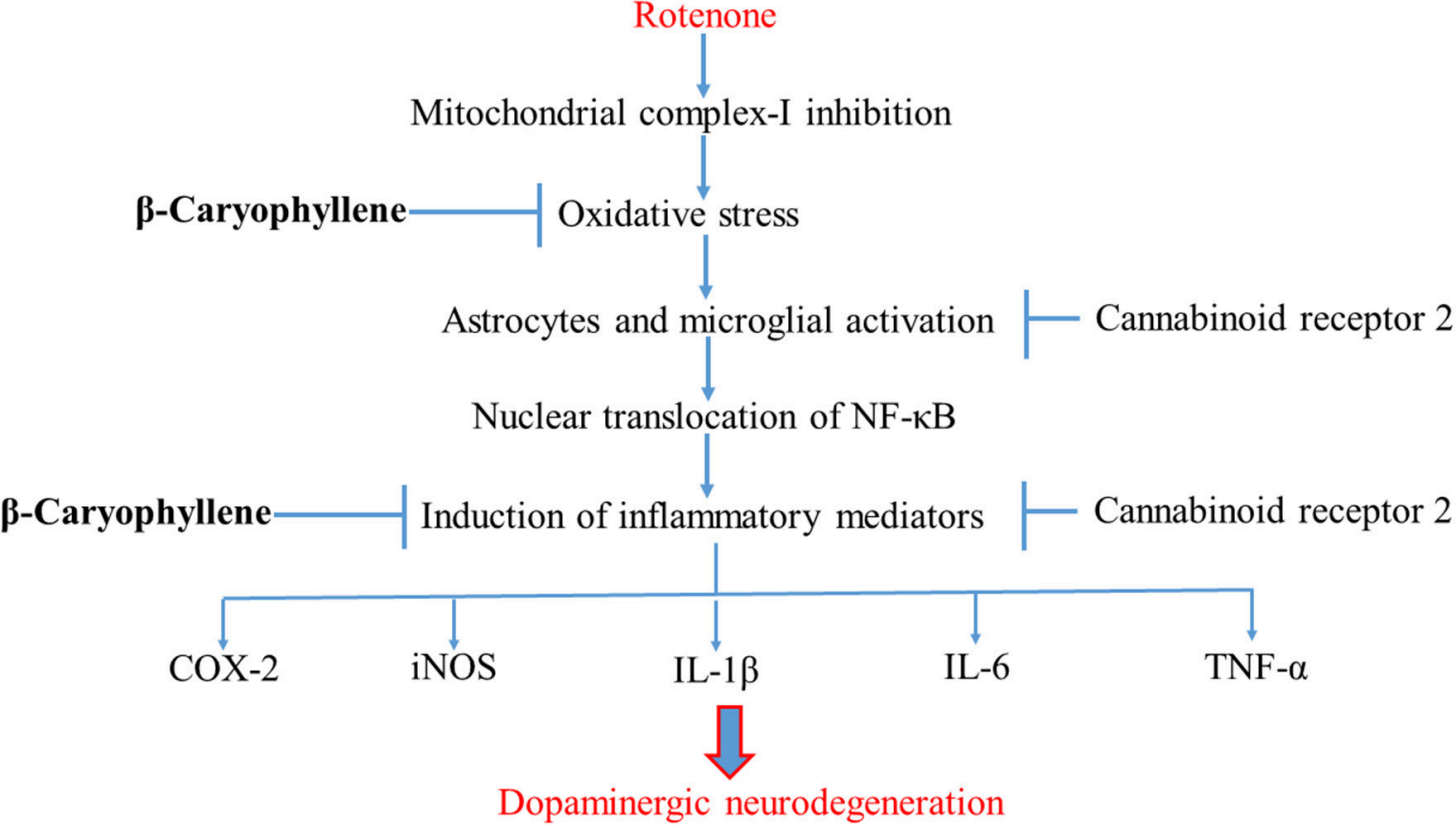
**Schematic representation of the neuroprotective mechanism of action of BCP**.

In conclusion, the present study demonstrates that the plant-derived, sesquiterpene compound, BCP, exhibits potent anti-inflammatory and antioxidant effects in the ROT-induced rat model of PD. The major underlying mechanism is the activation of CB_2_ receptors, which results in attenuation of oxidative/nitrosative stress and neuroinflammation, inhibition of gliosis and pro-inflammatory cytokine release, and decline in the nigrostriatal degeneration. A diagram of the proposed underlying mechanism has been shown in Figure 8. Our findings clearly demonstrate that CB_2_ receptors may be an attractive therapeutic target for PD and BCP could be an attractive candidate molecule for the treatment of PD. Furthermore, BCP may be the first CB_2_ receptor agonist and plant derived cannabinoid to be developed in the quest for novel compounds that might improve conventional therapies as well as provide novel disease-modifying agents.

## Author contributions

All the authors provided important intellectual input, reviewed the content, and approved the final version of the manuscript. HJ, ME, SO: contributed significantly, conceived and designed the experiments, analyzed the data, read, wrote, and approved the manuscript. SA, HJ: performed the experiments. ME, SO: contributed reagents/materials/analysis tools. All the authors are agreed to be accountable for all aspects of the work in ensuring that questions related to the accuracy or integrity of any part of the work are appropriately investigated and resolved.

### Conflict of interest statement

The authors declare that the research was conducted in the absence of any commercial or financial relationships that could be construed as a potential conflict of interest.
